# Recombinant Vaccine Strain ASFV-G-Δ9GL/ΔUK Produced in the IPKM Cell Line Is Genetically Stable and Efficacious in Inducing Protection in Pigs Challenged with the Virulent African Swine Fever Virus Field Isolate Georgia 2010

**DOI:** 10.3390/pathogens13040319

**Published:** 2024-04-13

**Authors:** Elizabeth Ramirez-Medina, Ayushi Rai, Nallely Espinoza, Edward Spinard, Ediane Silva, Leeanna Burton, Jason Clark, Amanda Meyers, Alyssa Valladares, Lauro Velazquez-Salinas, Cyril G. Gay, Douglas P. Gladue, Manuel V. Borca

**Affiliations:** 1 U.S. Department of Agriculture, Agricultural Research Service, Foreign Animal Disease Research Unit, Plum Island Animal Disease Center, Orient, NY 11957, USA; elizabeth.ramirez@usda.gov (E.R.-M.); ayushi.rai@usda.gov (A.R.); nallely.espinoza@usda.gov (N.E.); edward.spinard@usda.gov (E.S.); amanda.meyers@usda.gov (A.M.); alyssa.valladares@usda.gov (A.V.); lauro.velazquez@usda.gov (L.V.-S.); 2 U.S. Department of Agriculture, Agricultural Research Service, Foreign Animal Disease Research Unit, National Bio and Agro-Defense Facility, Manhattan, KS 66502, USA; ediane.silva@usda.gov (E.S.); leeanna.burton@usda.gov (L.B.); jason.clark@usda.gov (J.C.); 3 Oak Ridge Institute for Science and Education (ORISE), Oak Ridge, TN 37830, USA; 4 U.S. Department of Agriculture, Agricultural Research Service, Beltsville, MD 20705, USA; cyril.gay@usda.gov

**Keywords:** ASFV, ASF, ASFV vaccine, ASFV-G-Δ9GL/ΔUK

## Abstract

We have previously reported that the recombinant African Swine Fever (ASF) vaccine candidate ASFV-G-Δ9GL/ΔUK efficiently induces protection in domestic pigs challenged with the virulent strain Georgia 2010 (ASFV-G). As reported, ASFV-G-Δ9GL/ΔUK induces protection, while intramuscularly (IM), administered at doses of 10^4^ HAD_50_ or higher, prevents ASF clinical disease in animals infected with the homologous ASFV g strain. Like other recombinant vaccine candidates obtained from ASFV field isolates, ASFV-G-Δ9GL/ΔUK stocks need to be produced in primary cultures of swine macrophages, which constitutes an important limitation in the production of large virus stocks at the industrial level. Here, we describe the development of ASFV-G-Δ9GL/ΔUK stocks using IPKM (Immortalized Porcine Kidney Macrophage) cells, which are derived from swine macrophages. We show that ten successive passages of ASFV-G-Δ9GL/ΔUK in IPKM cells induced small changes in the virus genome. The produced virus, ASFV-G-Δ9GL/ΔUKp10, presented a similar level of replication in swine macrophages cultures to that of the original ASFV-G-Δ9GL/ΔUK (ASFV-G-Δ9GL/ΔUKp0). The protective efficacy of ASFV-G-Δ9GL/ΔUKp10 was evaluated in pigs that were IM-inoculated with either 10^4^ or 10^6^ HAD_50_ of ASFV-G-Δ9GL/ΔUKp10. While animals inoculated with 10^4^ HAD_50_ present a partial protection against the experimental infection with the virulent parental virus ASFV-G, those inoculated with 10^6^ HAD_50_ were completely protected. Therefore, as was just recently reported for another ASF vaccine candidate, ASFV-G-ΔI177L, IPKM cells are an effective alternative to produce stocks for vaccine strains which only grow in swine macrophages.

## 1. Introduction

African Swine Fever virus (ASFV) produces a lethal disease in domestic swine. African Swine Fever (ASF), which severely affects the production of domestic pigs worldwide, is currently widely distributed, being present in Africa, Asia, Europe, and, recently, in the Caribbean region [1,2,3]. ASFV is a large and structurally complex virus. Its genome is composed of double-stranded DNA of approximately 180–190 kilobase pairs which encode more than 160 genes [4,5,6].

Although the development of vaccines to prevent ASF was attempted for years [7], commercial vaccines were not available until very recently. Therefore, the control of the disease was based on the elimination of infected and susceptible animals, as well as the restricted mobility of infected herds [8,9].

The use of recombinant attenuated virus strains as potential vaccine candidates has recently increased [10,11,12]. Novel vaccine candidates were developed by deleting ASFV genes involved in the production of the disease in pigs. In general, these recombinant vaccine strains were efficacious in protecting pigs against the challenge with the homologous virulent field isolate [13,14,15,16,17,18,19,20,21,22].

Among those recombinant live attenuated viruses is the ASFV-G-Δ9GL/ΔUK [23]. ASFV-G-Δ9GL/ΔUK was developed by a double gene deletion in the genome of the virulent isolate Georgia 2010 (ASFV-G) of the 9GL and the UK genes. This recombinant virus was shown to have no residual virulence in domestic pigs even when parenterally inoculated at doses as high as 10^6^ HAD_50_ [23]. Importantly, ASFV-G-Δ9GL/ΔUK efficaciously induces protection in vaccinated pigs against the experimental infection using the highly virulent homologous ASFV g [23]. ASFV-G-Δ9GL/ΔUK was reported to produce protection as early as 2 weeks after a single vaccine dose of at least 10^4^ HAD_50_ [23]. ASFV-G-Δ9GL/ΔUK was developed, purified, and stock was generated using primary cell cultures of swine macrophages. The use of primary cell cultures is a difficulty in the necessary scaling up of vaccine production with commercial purposes. As an alternative, the adaptation of ASFV to grow in a cell line is usually accompanied by significant and undesirable modifications in the virus genome that appear during the process of the virus adaptation [24]. Here, we report the production of ASFV-G-Δ9GL/ΔUK stocks utilizing the cell line IPKM as growing substrate [25]. It is demonstrated that ASFV-G-Δ9GL/ΔUK replicates efficiently in IPKM cells incorporating minimal genomic changes. In addition, pigs were partially and fully protected against the challenge with the virulent parental virus when they received a single vaccine dose containing 10^6^ or 10^4^ HAD_50_ of ASFV-G-Δ9GL/ΔUK, respectively. Consequently, IPKM cells can be an option for the massive production of ASFV-G-Δ9GL/ΔUK stocks.

## 2. Materials and Methods

### 2.1. Viruses and Cells

The ASFV-G-Δ9GL/ΔUK vaccine was produced in the Plum Island Animal Disease Center, as previously reported [13]. Primary cultures of peripheral blood swine macrophages were produced, as reported earlier [22]. Macrophages were used at a final concentration of 5 × 10^6^ cells/mL to coat 96 or 6 well plates, as needed. Titrations of ASFV-G-Δ9GL/ΔUK and its parental field isolate ASFV g were implemented in primary swine macrophage cultures as earlier reported [22] and summarized below. The immortalized porcine kidney macrophage derived cell line IPKM [25] was kindly provided by Dr Kokuho Takehiro from the National Institute of Animal Health of Japan. IPKM cells were grown under culture conditions, as previously described [25]. ASFV-G-Δ9GL/ΔUK (ASFV-G-Δ9GL/ΔUKp0) and the parental ASFV g (ASFV-Gp0) were sequentially passed 10 times in IPKM cultures (MOI = 1), producing ASFV-G-Δ9GL/ΔUKp10 and ASFV-Gp10, respectively. Every passage proceeded until cytopathic effect reached approximately 80% of the cells. Intermediate virus stocks were prepared by one freezing and thawing step, the clarification of the obtained cell suspension by centrifugation, and their titration on primary swine macrophage cultures, as described below. Growth kinetics studies comparing different ASFV-G-Δ9GL/ΔUKp0 and p10 stocks and those of ASFV-Gp0 and p10 were performed at a MOI of 0.01 HAD_50_ using previously published protocols [22]. Virus yields were titrated at different times post-infection on swine macrophages. The presence of virus-infected cells was evaluated by hemadsorption (HA), as previously reported, and determined by the Reed and Muench method [26].

### 2.2. Sequencing and Analysis of the ASFV-G-∆I177L Genome

The procedures to obtain the full genomic sequence of ASFV-G-Δ9GL/ΔUKp0 and ASFV-G-Δ9GL/ΔUKp10 were exactly those recently reported [27].

### 2.3. Evaluation of ASFV-G-Δ9GL/ΔUKp10 Efficacy in Domestic Pigs

The ability of ASFV-G-Δ9GL/ΔUKp10 to protect domestic pigs against infection with the highly virulent ASFV g was assessed in 35–40 kg crossbreed pigs. Groups of 5 animals were inoculated intramuscularly (IM), receiving either 10^4^ or 10^6^ HAD_50_ of ASFV-G-Δ9GL/ΔUKp10. Another group of pigs was mock-inoculated with the culture medium. The presence of the ASF clinical disease as well as changes in body temperature reads were recorded daily for 28 days. The harvesting of blood samples (in heparinized blood collection tubes) to quantify the presence of the virus was scheduled to be performed at days 0, 4, 7, 11, 14, 21, and 28 post-infection (pi). By day 28 pi, all groups of pigs were IM-challenged with 10^2^ HAD_50_ of ASFV-G. Animals were monitored and sampled, as described above, until day 21 post challenge (pc). The experiments with animals were performed under biosafety level 3 conditions in the animal facilities at Plum Island Animal Disease Center, strictly following a protocol approved by the Institutional Animal Care and Use Committee (225.06-19-R_090716, approved on 9 June 2019).

## 3. Results and Discussion

### 3.1. Effect of Sequential Passages of ASFV-G-Δ9GL/ΔUK in IPKM Cells

In most cases, the growth of an ASFV strain in a cell line requires a process of adaptation, usually accompanied by important genomic changes. To evaluate the capability of IPMK cells to allow the growth of ASFV-G-Δ9GL/ΔUK without a preliminary period of adaptation, ASFV-G-Δ9GL/ΔUK was subjected to a set of 10 successive passages in IPMK cells. In these passages, infection was performed using an MOI of 1 (the initial and all intermediate stocks’ titers were calculated based on titrations implemented in swine macrophages).

All virus passages in IPKM cells were halted when the cytopathic effect reached approximately 80% of the cells. At that point, cultures were frozen and each of the intermediate stocks prepared, as described in Materials and Methods. A similar experiment was also performed in parallel with the parental virus ASFV-G.

**Figure 1:**
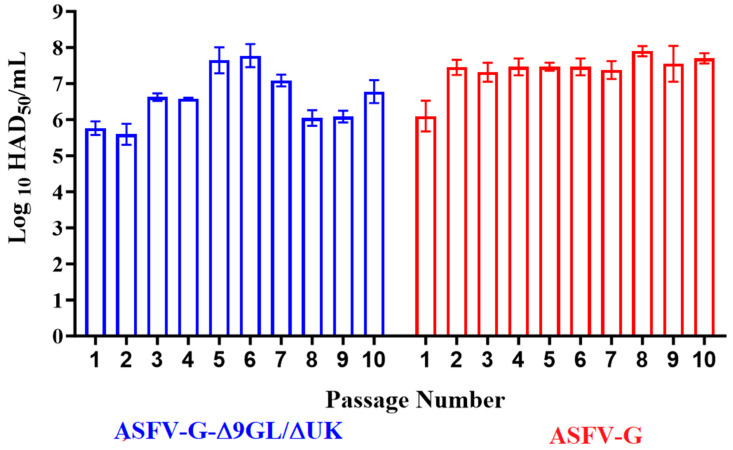
Virus yields of ASFV-G-Δ9GL/ΔUK in sequential passages in IPKM cells. ASFV-G-Δ9GL/ΔUK and ASFV g were sequentially passed 10 times (MOI = 1) in IPKM cell cultures. Viral titers in each passage were evaluated in primary swine macrophages and values expressed as HAD_50_/mL. Data represent averages and SD of two experiments.

The results showed that the virus yields of the recombinant ASFV-G-Δ9GL/ΔUK as well as ASFV g remained without large fluctuations during the 10 passages in the IPKM cells (Figure 1). Virus yield along the passages remained within a range, with titer values of 10^5.4–7.3^ HAD_50_/mL for ASFV-G-Δ9GL/ΔUK and between 10^5.8^ to 10^8^ HAD_50_/mL for ASFV-G. Therefore, the two viruses effectively replicate in the IPKM cells without needing a clear phase of adaptation. These data results support results already reported, showing that several ASFV isolates (Armenia07, Kenya05/Tk-1, Espana75 and Lisbon60) easily replicate in IPKM cells [28], reaching virus yields similar to those obtained in the primary cultures of swine macrophages.

### 3.2. Genomic Stability of ASFV-G-Δ9GL/ΔUK in IPKM Cells

To assess the genomic stability of ASFV-G-Δ9GL/ΔUK during the 10 successive passages in IPKM cells (ASFV-G-Δ9GL/ΔUK p10), ASFV-G-Δ9GL/ΔUKp10 was sequenced and the result was compared to that of the ASFV-G-Δ9GL/ΔUKp0. Just one mutation of high confidence (over 70% of the reads at that position contained the SNP) was observed, at nucleotide position 127,166 within the CP530R gene, where a G-to-A mutation produced a Gly-to-Ser substitution. This gene encodes for the virus polyprotein pp62, described as being essential to the processes of virus core development [29]. It is not clear if the glycine-to-serine amino acid substitution detected in the ASFV-G-Δ9GL/ΔUKp10 may affect the function of pp62L protein. Similar results in terms of the genome stability of ASFV strains sequentially passed in IPKM cells have been reported for Armenia 2007, ASFV g isolates and the vaccine candidate ASFV-G-ΔI177L [27,28].

### 3.3. Evaluation of the Virus Replication of the Recombinant ASFV-G-Δ9GL/UK in IPKM Cells

The growth kinetics of ASFV-G-Δ9GL/ΔUKp10 on IPKM cells was assessed by comparison with that of the original parental virus stock, ASFV-G-Δ9GL/ΔUKp0. In addition, the replication ability of both ASFV-Gp0 and ASFV-Gp10 was assessed. The study was conducted as a multistep growth assay on primary swine macrophage cultures. Infections were performed (MOI = 0.01) and virus yields assessed at 2, 24, 48, 72, and 96 h pi by titration performed in swine macrophages.

**Figure 2:**
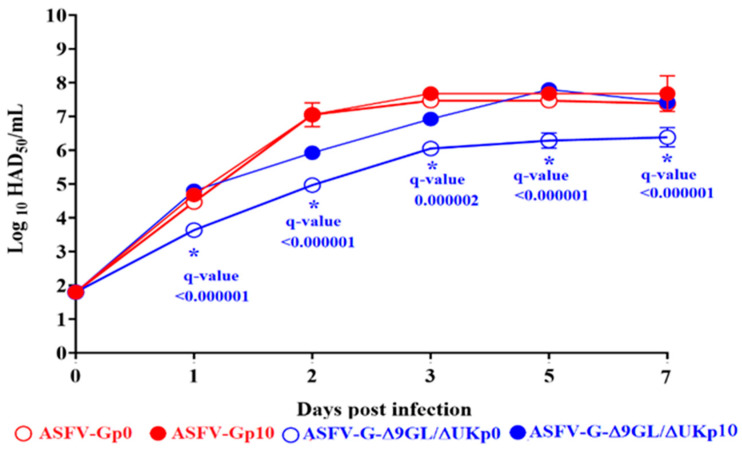
In vitro growth kinetics of ASFV-G-Δ9GL/ΔUkp10 in primary swine macrophages (MOI = 0.01). Samples were taken at the indicated time points and titrated in swine macrophages. Titrations were performed in swine macrophages. Data represent means and standard deviations from two independent experiments. The sensitivity of virus detection is ≥log10 1.8 HAD_50_/mL. The symbol (*) indicates significant differences between ASFV-G-Δ9GL/ΔUKp0 and ASFV-G-Δ9GL/ΔUKp10 at specific time points. Differences were inferred by the unpaired *t* test using the two-stage set up (Benjamine, Krieger and Yekutieli) method. The reliability of multiple comparisons was evaluated by the false discovery rate method (FDR), considering a q-value < 0.05.

As previously reported, ASFV-Gp10 presented (at MOI = 0.01) growth kinetics very similar to the original virus stock (ASFV-Gp0). (Figure 2). On the other hand, ASFV-G-Δ9GL/ΔUKp0 showed lower virus yields than ASFV-G-Δ9GL/ΔUKp10. In fact, ASFV-G-Δ9GL/ΔUKp10 exhibited growth kinetics similar to those of ASFV-Gp0 and ASFV-Gp10 viruses. Virus yield differences between ASFV-G-Δ9GL/ΔUKp0 and ASFV-G-Δ9GL/ΔUKp10 ranged between 10^1.5^ to 10^2.5^ HAD_50_/mL, regarding the evaluated sample time point post-infection. It is not clear if these differences could be completely justified by the small genomic changes acquired during the successive passages in IPKM cells.

### 3.4. ASFV-G-Δ9GL/ΔUKp10 Replication in Experimentally Infected Domestic Pigs

**Figure 3:**
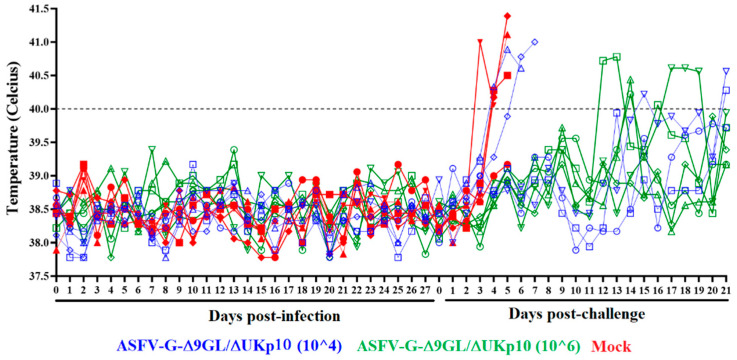
Body temperature in pigs (n = 5) IM inoculated (or Mock inoculated) with either 10^4^ or 10^6^ HAD_50_ of ASFV-G-Δ9GL/ΔUKp10 and challenged 28 days later with 10^2^ HAD_50_ of parental virulent ASFV-G. Data represent individual animals.

To assess the ability of ASFV-G-Δ9GL/ΔUKp10 to replicate in domestic pigs and efficaciously protect them against infection with the virulent ASFV g isolate, two groups (n = 5) of 35–40 kg pigs were IM-inoculated with either 10^4^ or 10^6^ HAD_50_. These doses of the vaccine were chosen since, when originally reported [23], ASFV-G-Δ9GL/ΔUK demonstrated the ability to protect against ASFV challenge. A group of similar characteristics was used as a control. The potential presence of clinical signs related to ASF was checked daily for 28 days after inoculation. Results demonstrated that all animal groups remained clinically normal for the whole observational period (Figure 3), indicating that ASFV-G-Δ9GL/ΔUKp10 continues to be fully attenuated when inoculated in domestic pigs, even at doses as high as 10^6^ HAD_50_.

**Figure 4:**
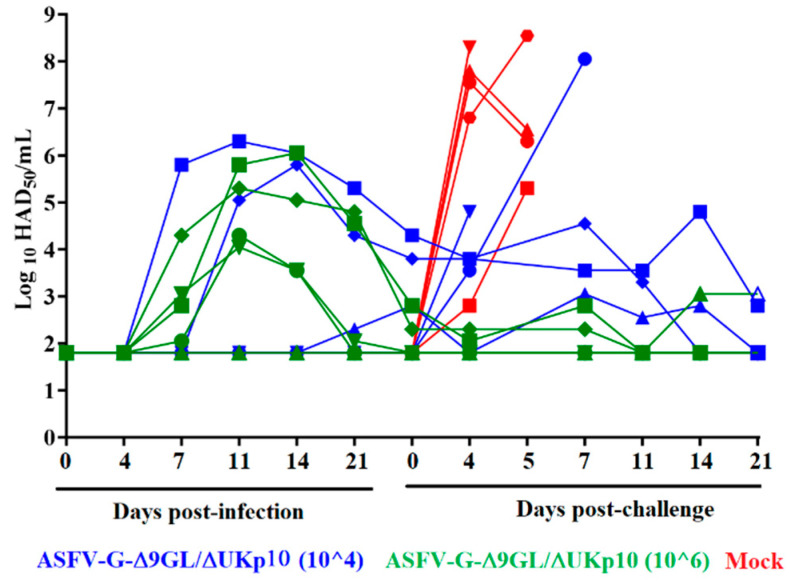
Viremias observed in pigs (n = 5) IM-inoculated (or Mock inoculated) with either 10^4^ or 10^6^ HAD_50_ of ASFV-G-Δ9GL/ΔUKp10 or mock-inoculated and challenged 28 days later with 10^2^ HAD_50_ of ASFV-G. Data represent individual animals. Sensitivity of virus detection: ≥10^1.8^ TCID_50_/mL.

The ability of ASFV-G-Δ9GL/ΔUKp10 to replicate after its inoculation was assessed by quantifying its viremia titers. The pattern of viremia in the inoculated animals was heterogeneous in both groups (Figure 4). In the group inoculated with 10^4^ HAD_50_/mL, two of the animals showed undetectable levels of viremia throughout the observational period of 28 days pi. Another animal remained negative until day 21 pi, when it showed low titers (10^2.3^ HAD_50_/mL) that remained at that level until the day of challenge (28 days pi). Viremia in the fourth animal inoculated with 10^4^ HAD_50_/mL remained undetectable until day 11 pi, when high titers were detected (10^5.05^ HAD_50_/mL), and it showed variable titers (ranging between 10^5.8–3.8^ HAD_50_/mL) until day 28 pi. The fifth animal in the group showed relatively high viremia titers (ranging between 10^4.3–6.3^ HAD50/mL) from day 7 pi until the challenge day.

With the exception of one pig, which presented undetectable viremia titers during the 28 days following inoculation, all animals receiving 10^6^ HAD_50_/mL of ASFV-G-Δ9GL/ΔUKp10 showed low-to-medium titers by day 7 pi (10^2.05–4.3^ HAD50/mL). Two of these animals evolved, showing medium-to-high viremia titers (ranging between 10^4.55–5.8^ HAD50/mL) by days 11 to 21 pi, and lower titers by the day of challenge (10^2.3–2.8^ HAD50/mL). The remaining two pigs of the group presented medium titers (10^2.05–4.3^ HAD50/mL) on days 11 and 21 pi and undetectable levels by day 28 pi.

These levels of replication are clearly lower than viremia titers detected in pigs IM-inoculated with similar doses of the original stock ASFV-G-Δ9GL/ΔUK (ASFV-G-Δ9GL/ΔUKp0) [23].

### 3.5. Evaluation of the Protective Effect of ASFV-G-Δ9GL/ΔUKp10 on Experimental Inoculation with the Parental ASFV-G

The ability of ASFV-G-Δ9GL/ΔUK to protect animals against the experimental infection with the parental virus ASFV g was evaluated by IM challenging the animals previously inoculated with ASFV-G-Δ9GL/ΔUK 28 days later with 10^2^ HAD_50_ of ASFV-G. The mock vaccinated control pigs were similarly infected.

**Figure 5:**
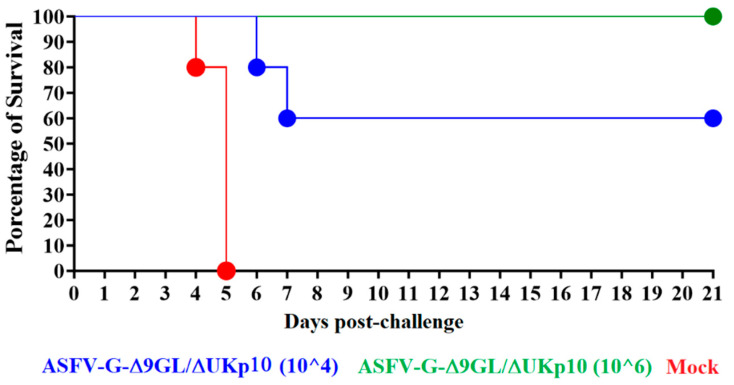
Mortality in animals (n = 5) IM-inoculated (or mock-inoculated) with either 10^4^ or 10^6^ HAD_50_ of ASFV-G-Δ9GL/ΔUKp10 and challenged 28 days later with 10^2^ HAD_50_ of ASFV-G.

The mock-inoculated pigs presented initial signs of ASF by days 3–4 post challenge (dpc), quickly getting worse with all of them euthanized due to the severity of the clinical signs by day 4, one of them early, and the remaining four on day 5 pc (Figure 3 and Figure 5). Conversely, all the animals inoculated with 10^6^ HAD_50_ of ASFV-G-Δ9GL/ΔUKp10 remained clinically normal during the observation period, except for two, presenting one period of transient mild body temperature elevation without any other additional sign associated with ASF (Figure 3 and Figure 5). Animals in the group receiving 10^4^ HAD_50_ of ASFV-G-Δ9GL/ΔUKp10 presented a heterogenous response after the challenge. Two of the animals presented initial signs of disease by days 4–5 post challenge, followed by a quick evolution to the severe clinical form of the disease and the need to be euthanized by days 6–7 pc. The remaining three animals did not show any clinical sign associated with ASF during the 21-day observational period, with the exception of very mild and transitory elevation in body temperature (Figure 3 and Figure 5).

Viremias in the mock-vaccinated animals increased after the challenge. In all but one animal, titers ranged between 10^6.5^ and 10^8.3^ HAD_50_/mL by day 4 pc and remained at that level until all the animals were euthanized. The remaining animal in this group presented a low (10^2.5^ HAD_50_) viremia by day 4 pc, increasing to 10^5^ HAD_50_ at the time of the euthanasia (Figure 4). Animals inoculated with 10^6^ HAD_50_ of ASFV-G-Δ9GL/ΔUKp10 presented low viremias after the challenge, ranging between 10^1.8^ and 10^3^ HAD_50_/mL. Viremia in most of these animals, except for one, decreased until it hit undetectable levels (≤10^1.8^ HAD_50_/mL) before the end of the 21-day pc observational period. Animals receiving 10^4^ HAD_50_ of ASFV-G-Δ9GL/ΔUKp10 had a viremia pattern in accordance with their clinical status. The three animals surviving the challenge presented low viremias after the challenge, ranging between undetectable (≤10^1.8^ HAD_50_/mL) and 10^2.8^ HAD_50_/mL. The two animals that succumbed to the challenge quickly presented medium-to-high viremias by the day they were euthanized because of the severity of the disease (10^4.5^ and 10^7.8^ HAD_50_/mL, respectively). Viremia in two of the remaining three animals decreased until it reached undetectable levels.

It is shown that the recombinant vaccine candidate ASFV-G-Δ9GL/ΔUK may be produced in the cell line IPKM. A stock virus produced after 10 sequential passages in these cells is still safe and efficacious in protecting animals against the infection with the virulent ASFV g when used at a dose of 10^6^ HAD_50_/mL. It is not clear why, though no main genetic changes are observed in the ASFV-G-Δ9GL/ΔUKp10 genome; this strain is slightly less efficacious than the parental ASFV-G-Δ9GL/ΔUK, which is able to protect pigs at doses of 10^4^ HAD_50_/mL.

As it was previously described for the ASFV-G-ΔI177L vaccine strain [27], these results demonstrate the feasibility of employing the IPKM cell line to produce ASFV vaccine strains, which were initially developed and grown in primary swine macrophage cell cultures, signifying a restriction in the manufacture of a vaccine with commercial purposes.

## Data Availability

All data are included in the manuscript.
